# The relationship between study strategies and academic performance

**DOI:** 10.5116/ijme.57dc.fe0f

**Published:** 2016-10-07

**Authors:** Yuanyuan Zhou, Lori Graham, Courtney West

**Affiliations:** 1 Office of Medical Education, Texas A&M University Health Science Center, College of Medicine, Bryan, Texas, USA

**Keywords:** Study strategies, learning strategies, assessment, academic performance

## Abstract

**Objectives:**

To investigate if and to what extent the Learning and Study Strategy Inventory (LASSI) and the Self-Directed Learning Readiness Scale (SDLRS) yield academic performance predictors; To examine if LASSI findings are consistent with previous research.

**Methods:**

Medical school students completed the LASSI and SDLRS before their first and second years (n = 168). Correlational and regression analyses were used to determine the predictive value of the LASSI and the SDLRS. Paired t-tests were used to test if the two measurement points differed. Bivariate correlations and R^2^s were compared with five other relevant studies.

**Results:**

The SDLRS was moderately correlated with all LASSI subscales in both measures (r_(152)_ =.255, p=.001) to (r_(152)_ =.592, p =.000). The first SDLRS, nor the first LASSI, were predictive of academic performance. The second LASSI measure was a significant predictor of academic performance (R^2^_(138)_ = 0.188, p = .003). Six prior LASSI studies yielded a range of R^2^s from 10-49%.

**Conclusions:**

The SDLRS is moderately correlated with all LASSI subscales. However, the predictive value of the SDLRS and LASSI differ. The SDLRS does not appear to be directly related to academic performance, but LASSI subscales: Concentration, Motivation, Time Management, and Test Strategies tend to be correlated. The explained LASSI variance ranges from 10% to 49%, indicating a small to substantial effect. Utilizing the LASSI to provide medical school students with information about their strengths and weaknesses and implementing targeted support in specific study strategies may yield positive academic performance outcomes.

## Introduction

Why do some students perform well academically in medical school while others do not? This question has been explored and investigated in a variety of ways. Pre-matriculation data such as MCAT scores and Undergraduate GPA (UGPA) have been used to predict success in medical school,^1^^,^^2^ and so have certain study/learning strategies.^3^^-^^20^

Identifying relationships between academic performance and study/learning strategies is important because it offers the opportunity to provide students with specific feedback related to strengths and weaknesses that can be used to help scaffold students’ learning.  Many relevant studies of pre-admission aptitude and study skills dealt with broad concepts and exhibited mixed results. For example, Sleight and Mavis created a Study Aid questionnaire and found high performers were less reliant on study aids.^21^ Lobb, Wilkin, McCaffrey, Wilson, and Bentley applied three commercial instruments and found that none of them assessed abilities directly related to academic performance.^22^ However, one of those instruments, the Learning and Study Strategy Inventory (LASSI) was utilized in West and Sadoski’s research and some of the LASSI subscales appeared to predict preclinical academic performance.^6^

Due to these findings, several questions emerged.  First of all, how should study/learning strategies be defined? In the existing literature, the LASSI was frequently used to measure students’ study/learning strategies. However, researchers, including those who utilized the LASSI, may not agree that its' ten domains encompass all “study strategies”. For example, self-directed readiness does not appear to be directly measured by the LASSI.  However, helping students develop into self-directed learners has been a focus in medical education since the Liaison Committee on Medical Education (LCME)^23^requires that programs “foster the skills necessary for lifelong learning.”

Based on Cook and West’s^24^ suggestions, a systematic review was conducted utilizing the following databases: PubMed, EMBASE, SCOPUS, PsycINFO, Web of Science, CINAHL, and ERIC  and keywords “study/learning strategy (strategies)”, “academic performance” and “LASSI”. Even though there were hundreds of articles, only 19 studies were relevant and were kept for further analyses (a comprehensive summary of the 19 studies are provided in the Appendix 1). The excluded articles included qualitative studies, quantitative studies that used advanced methods to investigate more complicated scenarios, or studies that contained the same keywords but were irrelevant. Of the 19 studies, most^3^^-^^11^^,^^13^^,^^14^^,^^16^^,^^17^^,^^19^^,^^20^ concluded that study strategies have an influence on academic performance as indicated by GPA^3^^,^^14^^,^^16^^,^^19^^,^^20^ school performance,^5^^-^^8^^,^^15^^,^^17^^,^^18^^,^^22^ or standardized exams4,^5^^,^^8^^,^^9^ (see Appendix 1). A retrospective review of those results was difficult due to several reasons. First, some studies’ analyses may have been inappropriate.  For example, converting a continuous variable into a categorical variable (e.g., defining academic performance as low, medium, or high) was common in the reviewed articles^3^^,^^4^^,^^7^^,^^8^^,^^15^^,^^18^^,^^25^. Another approach used percentile scores in the multiple regression models.^22^ However, percentile scores are not interval or ratio scaled and should not be utilized. Many methodologists^26^^,^^27^ have suggested caution when utilizing these types of practices because they may damage the nature of the relationship and weaken the conclusions. Second, the discrepancies of analytical methods and models between studies did not facilitate a direct comparison. Multiple regression was the most common method, but ANOVA^8^^,^^20^, ANCOVA^16^^,^^25^, correlational analysis,^9^^,^^19^ and logistic regression^12^ was also utilized. Even those who adopted the same methods like multiple regression, used different predictors,^5^^,^^6^^,^^17^^,^^18^^,^^22^ or predictors with different scales (i.e.,continuous scales^5^^,^^6^^,^^10^^,^^11^^,^^14^^,^^17^^,^^22^^,^^28^ vs. categorical scales^4^^,^^18^^,^^29^ or different forms (i.e., original subscale scores^4^^-^^6^^,^^10^^,^^14^^,^^17^^,^^18^ vs. latent scores).^11^^,^^13^  The beta weights were also not comparable between studies. Third, most studies failed to report the key components such as correlation matrices and standard deviations as suggested by Zientek and Thompson.^30^ This limited cross comparisons and retrospective thinking. Fourth, the narrow definition of learning strategies, such as those which only included the domains measured by LASSI, restricted generalizability of the results. Similarly, how academic performance was defined (such as course grade, GPA, or standardized test scores) may have also influenced the results. Last, but not least, most studies only addressed whether or not there was an effect of study strategies on academic performance, but did not address to what extent the study strategies may affect academic performance. Even if there were many positive conclusions (i.e., yes, there was an effect), this would not lead us closer to estimating the magnitude of the effect.  The size of the effect is a more important indicator of the potential importance of using the LASSI in medical school.

Due to the listed limitations, we redesigned the study and re-conducted the research project in 2013. There were two primary changes.  First, in addition to the LASSI, we added the Self-Directed Learning Readiness Scale (SDLRS)^31^ as another measure due to the increasing emphasis on self-directed learning in medical schools.  The SDLRS was selected because it is the most widely used assessment to measure self-directed learning readiness,^32^ and we wanted to assess how learning readiness influenced student performance on specific tasks.^33^^-^^35^ We also added another measurement point at the beginning of the second year (i.e., 2014), so we could examine the difference between the two measurements and the effect related to timing.

The purpose of this study was to compare LASSI and SDLRS outcomes and to determine if the measures yield academic performance predictors.  The research questions are:

   •   Does the LASSI and the SDLRS measure the same construct?

   •   Are LASSI and SDLRS scores predictive of academic performance?  If so, is one measure more valuable than another?

   •   Assuming the LASSI or the SDLRS are good predictors, how much variance of academic performance can be explained by LASSI or SDLRS scores?

The authors integrated current study data with published relevant research^5^^,^^6^^,^^10^^,^^14^^,^^17^ and conducted a secondary analysis of the two articles that contained a bivariate correlation matrix and related descriptive statistics.^5^^,^^6^ The aim of the integration was to find a more generalizable index (i.e., R^2^) other than null hypothesis testing that may enable educational practitioners to estimate the size of the effect of study strategies on preclinical academic performance.

## Methods

### Participants

The Institutional Review Board at Texas A&M University approved the study. A sample of 168 (67 males, 74 females, and 27 not indicated) out of 203 medical students at Texas A&M Health Science Center College of Medicine voluntarily participated in the study and signed consent forms. Students received access information for the LASSI and SDLRS during orientation and were given time during the session to complete the two learning assessments.  The sessions were held at the beginning of their first year of medical school and again at the beginning of their second year. Seven students did not complete the first LASSI, and 13 students did not finish the first SDLRS assessment. The numbers of students who did not complete the second LASSI and SDLRS were 20 and 58, respectively.

**Table 1:** Pre- and Post- Inter-Item Correlations, Means, and Standard deviation (N = 168)

Items	1	2	3	4	5	6	7	8	9	10	11	12	13	14	Post Mean	Post SD
1 Academic performance	––	.248^**^	.250^**^	.123	.113	-.032	.173^*^	.128	.218^**^	.141	.278^**^	-.130	.213^*^	.249^**^	664.52	38.08
2 MCAT	.212^*^	––	.090	-.185	.342^**^	-.281^**^	-.166	-.004	-.218^*^	-.099	.166	-.241^**^	-.253^**^	.116	28.79	3.04
3 UGPA	.308^**^	.090	––	-.008	.068	.041	.087	.108	.120	.054	.122	.090	.120	.149	3.63	.25
4 SDLRS	-.015	-.034	-.270^**^	––	.276^**^	.505^**^	.429^**^	.494^**^	.437^**^	.289^**^	.517^**^	.322^**^	.380^**^	.474^**^	231.47	24.56
LASSI subscales																
5 Anxiety	.038	.260^**^	-.108	.446^**^	––	.138	.217^**^	.361^**^	.111	.078	.474^**^	-.155	.089	.531^**^	29.77	6.54
6 Attitude	-.100	-.263^**^	-.056	.438^**^	.281^**^	––	.489^**^	.260^**^	.498^**^	.304^**^	.329^**^	.493^**^	.391^**^	.317^**^	32.68	4.05
7 Concentration	.023	-.087	-.106	.369^**^	.424^**^	.483^**^	––	.287^**^	.497^**^	.399^**^	.343^**^	.232^**^	.533^**^	.417^**^	26.51	5.96
8 Information processing	-.058	-.024	-.115	.572^**^	.367^**^	.303^**^	.325^**^	––	.351^**^	.392^**^	.448^**^	.149	.171^*^	.434^**^	28.93	4.68
9 Motivation	.085	-.242^**^	.093	.371^**^	.188^*^	.423^**^	.356^**^	.279^**^	––	.475^**^	.446^**^	.334^**^	.673^**^	.519^**^	32.01	4.13
10 Self-Testing	.043	-.241^**^	-.028	.348^**^	.032	.234^**^	.378^**^	.414^**^	.391^**^	––	.355^**^	.343^**^	.444^**^	.393^**^	24.29	5.35
11 Selecting Main Idea	.104	.056	-.069	.592^**^	.568^**^	.294^**^	.545^**^	.411^**^	.309^**^	.276^**^	––	.153	.253^**^	.745^**^	29.21	5.74
12 Study Aid	-.125	-.371^**^	-.043	.264^**^	-.083	.344^**^	.319^**^	.349^**^	.392^**^	.460^**^	.099	––	.331^**^	.030	23.45	4.73
13 Time Management	-.023	-.258^**^	.090	.255^**^	.080	.302^**^	.506^**^	.330^**^	.543^**^	.596^**^	.285^**^	.519^**^	––	.288^**^	28.76	6.16
14 Test Strategies	.172^*^	.089	-.030	.484^**^	.578^**^	.390^**^	.556^**^	.365^**^	.383^**^	.199^*^	.763^**^	.076	.266^**^	––	30.17	4.92
Pre Mean	414.21	28.79	3.63	239.68	29.66	33.99	28.52	30.35	34.92	25.36	29.71	25.57	28.91	31.63		
Pre SD	24.55	3.04	.25	21.78	6.39	3.35	5.78	5.13	3.66	5.86	5.43	5.94	6.69	4.38		

*Correlation is statistically significant at the 0.05 level (2-tailed). **Correlation is statistically significant at the 0.01 level (2-tailed). Key: SD = standard deviation; SDLRS = Self-Directed Learning Readiness Scale; LASSI = Learning and Study Strategies Inventory. The lower left triangle was the bivariate correlation matrix for students Year I overall performance, MCAT, UGPA, pre-SDLRS, and pre-LASSI subscales, and the upper triangle was the bivariate correlation matrix for students Year II overall performance, MCAT, UGPA, post-SDLRS, and post-LASSI subscales.

The researchers also conducted secondary analyses on the only two studies^5^^,^^6^ that contain sufficient information to  enable secondary analyses. Three other studies^10^^,^^14^^,^^17^ that reported partial information are also included in the summary table.

### Data collection

The LASSI^36^ includes 80 items rated on a five-point Likert-type scale.  Immediately upon completion, each participant receives a performance profile including 10 subscales: Anxiety, Attitude, Concentration, Information Processing, Motivation, Selecting Main Idea, Self-Testing, Study Aids, Test Strategies, and Time Management. The SDLRS  is a valid assessment^37^ consisting of 58 items related to attitudes, skills, and characteristics to determine one’s current level of self-directed learning readiness.^31^  The SDLRS scores range from 58 (below average) to 290 (above average) with an average score of 214.^31^

Academic Performance Data included Medical College Achievement Test (MCAT), UGPA, Year I average, and Year II average. The MCAT overall score was utilized as well as overall UGPA.  The Year I average included performance in integrated courses including Gross Anatomy, Histology, Biochemistry, Genetics, Cell Physiology, and Pharmacology, as well as Introduction to Disease and Neuroscience.  The Year II average included performance in Hematology/Oncology, Cardiovascular, Respiratory, Renal/Genitourinary, Gastrointestinal/Nutrition, Endocrinology/Reproductive Science/Human Sexuality, and Integument/Musculoskeletal organ system blocks.

### Data analysis

SPSS 22.0 was used to conduct the statistical analyses. Descriptive statistics such as means and standard deviations were calculated. A correlational analysis including scatterplots was conducted to understand the relationship between the LASSI and SDLRS and the instruments’ predictive validity. Regression analyses were utilized to determine the predictive value of the LASSI and SDLRS. Pairwise deletion strategy was selected due to its’ ability to handle missing data. To investigate the change in LASSI and SDLRS over the course of the first year of medical school, we utilized paired t-tests to analyze mean changes.  Further exploration of the LASSI included comparing correlations and R^2^ s for six studies. 

## Results

Table 1 includes means, standard deviations, and bivariate corelation coefficients for all targeted variables. The SDLRS was significantly correlated with all LASSI subscales in both the first and the second measure: the correlations ranged from r_(__152)_ = .255, p = .001 to r_(152)_ = .592, p = .000 for the first measure and from r_(105)_ = .276, p=.004 to r_(105)_ = .517, p = .000.

Scatterplots also confirmed the linear bivariate positive correlation between the SDLRS and each LASSI subscale. The subscales “Selecting Main Idea” and “Information Processing” had the highest correlation with SDLRS in both measures.  Academic performance was always statistically significantly correlated with the MCAT and UGPA.  For Year I overall performance, the association with MCAT was r_(__126)_ = .212, p < 0.05 and the association with UGPA was r_(130)_ = .308, p < 0.01. For Year II overall performance, the association with MCAT was r_(__130)_ = .248, p < 0.01 and the association with UGPA was r_(135)_ = .250, p < 0.01.

When comparing the first and the second measures, the authors discovered scores on the LASSI for all ten subscales and SDLRS decreased. To detect to what extent the two measures changed, ten paired t-tests on the first and second LASSI subscales and one paired t-test for the first and second SDLRS tests were conducted. Results indicated that for the LASSI, eight subscales significantly decreased. They were: Attitude (Cohen’s d_(147) _= .37, p < .001), Concentration (Cohen’s d_(147)_ = .38, p < .001), Information Processing (Cohen’s d_(147)_ = .32, p < 0.001), Motivation (Cohen’s d_(147) _= .75, p < .001), Self-Testing (Cohen’s d_(147)_ = .18, p = .028), Study Aids (Cohen’s d_(147)_ = .37, p < .001), and Test Strategies (Cohen’s d_(147) _= .31, p < .001). The score for the SDLRS also significantly decreased in the post measure (Cohen’s d_(__104) _= .42, p = .000). After the drop, the LASSI subscales’ scores were more closely associated with academic performance.  However, the change in scores on the second test did not make the SDLRS a better predictor.

**Table 2:** Regression Models Used to Determine if Learning Strategies Predict Academic Performance (N = 168)

Model		R^2^	Adjusted R^2^		Change Statistics			
					R^2^ Change		F Change	p
Pre-matriculation								
	1	.090 (.109)	.075 (.094)		.090		6.013	.003
	2	.119 (.117)	.098 (.101)		.119		5.787	.004
Pre-matriculation With SDLRS								
	3	0.093		.070		.003	.348	.556
	4	.125		.094		.006	.628	.430
SDLRS only								
	5	.000		-.007		.000	.033	.855
	6	.015		.005		.015	1.555	.215
Pre-matriculation With LASSI								
	7	0.183		.098		.075	1.053	.404
	8	.228		.136		.111	1.442	.173
LASSI only								
	9	.096		.032		.096	1.508	.142
	10	.188		.123		.188	2.895	.003

Note: when pairwise deletion strategy was used to handle the missing data, the estimate of R2 has slight difference as new predictor(s) was added; Model 1- Outcome variable: Year I average; Predictors: MCAT, UGPA; Scores outside of parentheses were the estimate of R2 that needed to be compared with Model 3; scores in parentheses were the estimate of R2 that needed to be compared with Model 7; Model 2- Outcome variable: Year II average; Predictors: MCAT, UGPA; Scores outside of parentheses were the estimate of R2 that need to be compared with Model 4; scores in parentheses were the estimate of R2 that needed to be compared with Model 8; Model 3- Outcome variable: Year I average; Predictors: MCAT, UGPA, and SDLRS pre-test; Model 4- Outcome variable: Year II average; Predictors: MCAT, UGPA, and SDLRS post-test; Model 5- Outcome variable: Year I average; Predictor: SDLRS pre-test; Model 6- Outcome variable: Year II average; Predictor: SDLRS post-test; Model 7- Outcome variable: Year I average; Predictors: MCAT, UGPA, and LASSI Pre-test subscales; Model 8- Outcome variable: Year II average; Predictors: MCAT, UGPA, and LASSI post-test subscales; Model 9- Outcome variable: Year I average; Predictors: LASSI pre-test subscales; Model 10- Outcome variable: Year II average; Predictors: LASSI post-test subscales.

The correlation between the LASSI subscales and students’ overall performance was not consistent in the first and second measures. For the first LASSI measure, only “Test Strategies” was positively correlated with Year I academic performance (r_(__155)_ = .172, p < .05). With the second LASSI measure, five subscales were statistically significantly correlated with Year II academic performance. They included: Concentration (r_(__139)_ = .173, p < .05), Motivation (r _(140)_ = .218, p < .01), Selecting Main Ideas (r_(139) _= .278, p < .01), Time Management (r_(139) _= .213, p < .05), and Test Strategies (r_(140) _= .249, p < .01).

Table 2 summarized ten regression models’ results with pre-matriculation variable(s) (MCAT and undergraduate GPA) and/or study strategy (LASSI and SDLRS) as predictors. The model started with pre-matriculation variables as predictors and then added the ten LASSI subscales or SDLRS to the pre-matriculation variables. The pre-matriculation variables were then removed but the LASSI or SDLRS were still used as predictors. The results show that MCAT and undergraduate GPA were statistically significant predictors for Year I academic performance (R^2^_(__131)_ = .090, p = .003; see Model 1) and Year II academic performance (R^2^_(126) _= .119, p = .004; see Model 4). With the existence of MCAT and UGPA, adding the SDLRS or LASSI as predictor(s) did not significantly increase the explained variance for Year I (see Models 3 and 5) or Year II (see Models 4 and 6). If MCAT and UGPA were not included, in the first measure, neither the SDLRS nor the LASSI is good predictor(s) (the increased explained variance (R^2^) ranged from .000 to .096; see Model 5 and 7). However, in the second measure, when MCAT and UGPA were excluded, the LASSI became a statistically significant predictor of student academic performance (Model 10: R^2^_(__136)_ = .188, p = .003). Although factor analysis could neither replicate the factor structure proposed by Weinstein and Palmer^38^ nor replicate the factor structure proposed by Cano,^13^ Olaussen and Braten,^39^ and Olejnick and Nist,^40^ the results suggest  that students’ motivation and components of strategic learning contribute to academic performance.

The authors compared the current study to the systematically reviewed studies.  Of those, only five provided information that enabled a cross-study comparison. The two that reported enough data for secondary analyses were coincidently conducted at the same institution. The comparable information for all six studies (including the present study) is summarized in Table 3. Even though the six studies have variations by nature, the correlation matrices were relatively robust across studies. Concentration, Motivation, Time Management and Test Strategies were likely to be significantly correlated with outcome variables that measured academic performance. Anxiety was correlated occasionally.

Table 3 also provided the R^2^ and adjusted R^2^ for the multiple regression models with the ten LASSI subscales as predictors and the academic performance variable as the outcome variable. Based on the R^2 ^reported in the six studies, 10-49% of the total variance could be explained by the LASSI subscales. This means that the effect of learning strategies on academic performance is at least small, but it could be substantial.

**Table 3:** A Summary of correlations and R2 between LASSI and academic performance in six studies

Study Source	Current study (N = 168)		West (2011) (N = 106)	West (2014) (N = 79)	Kellogg (2010) (N = 65)	Lipsky (1999) (N = 442)	Loong (2012) (N = 156)		
Academic performance	Year I final	Year II final	Year 1 final	Step 1	Astronomy course grade	Year I GPA	Math performance		
							All	Home	International
Anxiety	0.04	0.11	0.07	.26^**^	0.516**	--	--	--	--
Attitude	-0.10	-0.03	.19^*^	0.04	0.00	--	--	--	--
Concentration	0.02	.173^*^	.23^**^	.28^**^	0.317*	--	--	--	--
Information processing	-0.06	0.13	0.12	-0.05	0.10	--	--	--	--
Motivation	0.09	.218^**^	.24^**^	0.12	0.535**	^‡^	--	--	--
Self-Testing	0.04	0.14	.36^**^	0.14	-0.16	--	--	--	--
Selecting Main Idea	0.10	.278^**^	0.08	0.14	0.11	--	--	--	--
Study Aid	-0.13	-0.13	.18^*^	-0.07	-0.21	--	--	--	--
Time Management	-0.02	.213^*^	.45^**^	.26^**^	0.24	^‡^	--	--	--
Test Strategies	.172^*^	.249^**^	.27^**^	0.17	0.24	--	--	--	--
R^2^	0.10^†^	0.19^†^	0.39^†^	0.19^†^	0.49	0.18	0.38	0.36	0.46
Adjusted R^2^	0.03^†^	0.12^†^	0.32^†^	0.07^†^	--	--	0.33	0.27	0.37

*Correlation is statistically significant at the 0.05 level (2-tailed). **Correlation is statistically significant at the 0.01 level (2-tailed). †The value was obtained from a secondary analysis. ‡ A good predictor but did not provide the value.

## Discussion

The SDLRS was statistically significantly correlated with the LASSI subscales. Someone who scores high on the SDLRS  is more likely to be able to: distinguish important information from less important information, connect new knowledge to prior knowledge, master preparation and test strategies, and have a positive attitude.^31^ However, the predictability of academic performance between the two scales was not equivalent.  Some LASSI subscales have higher correlation coefficients with academic performance than the SDLRS. Students who scored high on the SDLRS did not always perform well academically. The finding is consistent with a prior study which concluded that readiness for self-directed learning may not be necessary for learning foundational knowledge.^33^ However, the LASSI appears to be a good predictor when undergraduate GPA and MCAT are not controlled. This may indicate that students with a higher GPA and MCAT score tend to have better learning strategies. Therefore, in a multiple regression analysis with GPA, MCAT, and LASSI all as predictors, the unique contribution from learning skills cannot be identified.

The results for the two measures have statistically significant differences. All scores decreased in the second measure.  The drop resulted in the LASSI subscales being better academic performance predictors. The second measure may be a more accurate reflection of the real situation because the first LASSI was administered before students started taking courses in medical school. Therefore, the initial results are more likely to reflect their success prior to medical school. After students started their medical education, study strategies were likely adjusted based on the requirements and pacing in the medical school environment. The difference between the two measures indicates that study strategies may be dynamic. Timing could also be a moderator that affects the estimate of the entire effect. Since the factor analysis for the LASSI did not replicate the factor structure proposed by previous research, those who utilize it need to be aware that the scale may lack some stability and may be vulnerable to the testing environment. However, the LASSI performance profile does enable students to see how they did on the ten subscales as compared to a norm group and then target those areas if improvement is needed. The retrospective review of the five relevant studies identified that Concentration, Motivation, Time Management and Test Strategies were likely to be correlated with academic performance, although the R^2^ explained by all LASSI subscales vary in different cases. Future work is required to investigate what causes the fluctuation of explained variance. Possible factors could be inconsistencies in how academic performance is defined, how study strategies are measured, timing, administration procedures, and the testing environment. We considered the possible administration effect since the first LASSI measure was administered during orientation which was consistent with the other two research projects conducted at the same institution.^5^^,^^6^ However, the correlation with academic performance in the current study is smaller than in the two previous studies.  Perhaps this is due to the fact that the SDLRS was added in the current study requiring students to spend more time in the testing environment which may have resulted in testing fatigue.

This study has several limitations. How to define and measure study strategies still cannot be fully addressed even after adding the SDLRS as a predictor. The results indicate large differences for the two measures, but with the current research design, we were not able to address the factors that may have caused the differences. The retrospective review ended up with only five useful articles even though hundreds of articles with the same keywords were accessed. Therefore, additional work is needed in the area of study strategies and academic performance.

## Conclusions

Based on the statistical analyses of current data, the review of the LASSI and SDLRS research, and the summary data for the six relevant studies, LASSI subscales were non-negligible predictors for medical student academic performance in the preclinical curriculum. This study not only addressed whether or not there is an effect of study strategies on academic performance, but also addressed to what extent study strategies affect academic performance. Since a sufficient number of previous studies were not available, a complete meta-analysis was not conducted. Even though this is a limitation, broadening the scope of this study has served to provide more generalizable conclusions. 

In summary, we suggest utilizing the LASSI in medical school in combination with pre-matriculation data since the LASSI consistently yields academic performance predictors.  Those who score low in Concentration, Time Management, Test Strategies, and Motivation should receive targeted support in these areas when they enter medical school.  Completing another LASSI at the beginning of the second year of medical school would enable students to continue to monitor their study strategies and focus on specific weaknesses related to those strategies which appear to be predictors. Instilling the practice of study strategy monitoring via the LASSI will likely improve students’ academic performance and may facilitate the development of self-directed learning skills that “foster life-long learning”.^23^

### Acknowledgements

This study was funded in part by the Southern Group on Educational Affairs (SGEA) region of the AAMC through the Research in Medical Education (RIME)/Medical Education Scholarship, Research, and Evaluation (MESRE) Grant.

### Conflict of Interest

The authors declare that they have no conflict of interest. 

## Supplementary material

Supplementary file 1 Appendix 1. Published studies regarding the relationship between LASSI and academic performance
