# Supplementary material for: The relationship between study strategies and academic performance
Source: Int J Med Educ. 2016 Oct 7;7:324–32. doi: 10.5116/ijme.57dc.fe0f (PMC5056023; doi:10.5116/ijme.57dc.fe0f)
Supplement: Supplementary file 1 — Appendix 1. Published studies regarding the relationship between LASSI and academic performance [file ijme-7-324-S1.pdf]

Appendix 1.

Published studies regarding the relationship between LASSI and academic performance

Author	Sample size	Predictors	Academic performance	Statistical technique	Findings
Schutz, Gallagher, Tepe[3]	57	LASSI[a]	GPA[b]	*t*-tests	Differences in LASSI subtest are associated with GPA.
Schutz, Dalton, Tepe[4]	102	LASSI[a]	NBCE Part 1[b]	Mantel-Haenszel trend test, Multiple regression	Anxiety, Concentration, Selecting Main Ideas, and Test Strategies were significant predictors of NBCE scores.
Lobb, Wilkin, McCaffrey, Wilson, Bentley[22]	405	LASSI[a], PCAT_Percentile, Science GPA	First-year Grade[a]	Multiple regression	The LASSI do not assess abilities that are directly related to academic performance.
West, Kurz, Smith, Graham[5]	79	LASSI[a], MCAT, UGPA, Year 1&2 grade, CBSE, Customized NBME	Step 1[a]	Multiple regression	Concentration was associated with the Step 1 licensing exam performance.
Yip[7]	236	LASSI[a]	Not clearly defined[b]	ANOVA	Attitude, Motivation, Scheduling, Self-testing, and Test strategies were predictors of academic performance.
Albertini, Kelly, Matchett[9]	437	LASSI[c], Noel-Levitz	Reading & Math scores[a]	Correlational analysis	Self-Regulation and Will Components accounted for the students' initial GPA.
Loong[10]	156	LASSI[a]	Math scores[a]	Multiple regression	Attitude and Self-Testing were significant predictors of home students' math performance; Attitude and Test Strategies were significant predictors of international students' math performance.
Moliterni, De Stasio, Carboni, Di Chiacchio[11]	412	LASSI[c]	sum of (UGPA, attended exams, and HiGPA[c]	Multiple regression	Organization and Self-Evaluation factor was the best predictor of academic performance.
Gatto[12]	133	LASSI[a]	Academic risk[b] (Yes/No)	Logistic regression	Study strategies did not predict academic risk.
Cano[13]	956	LASSI[c]	Not clearly defined[b]	Multiple regression	Affective Strategies and Goal Strategies were positively linked to academic performance.
Yip, Chung[8]	218	LASSI[a]	Matriculation performance[b]	ANOVA	There were significant differences between study habits of students with high academic achievement and those with low academic achievement in Matriculation.
Lipsky[14]	442	LASSI[a]	Year I GPA[a]	Multiple regression	Motivation, Time Management, and Attitude were stronger predictors of first year GPA.
Gilles[15]	103	LASSI[b]	Final Course Grade[b]	Log linear regression	Log linear analyses fail to establish any relationship between LASSI and final course performances.
Ickes, Fraas[16]	59	LASSI[b], HGPA, ACT	Semester I & II GPA[a]	ANCOVA	Gains in study skills had little impact on academic performance.

Continued Appendix 1.

Author	Sample size	Predictors	Academic performance	Statistical technique	Findings
West, Sadoski[6]	106	LASSI[a], MCAT, UGPA	Year I final, Year I written, Year I practical, Year I customized NBME	Multiple regression	Time Management and Self-Testing were generally stronger predictors of first-semester academic performance.
Kellogg, Durben, Ayars-Junek[16]	65	LASSI[a], VARK, ADT, gender, age, previous academic performance	Astronomy Course grade[a]	Step-wise regression analysis	Anxiety and Motivation were good predictors of academic performance.
Franciosi[18]	108	LASSI[a], PSI	Not clearly defined[b]	Multiple regression	No significant relationship among self-reported problem-solving abilities, self-reported learning strategies, and academic achievement.
Hanlon O'Connell[19]	112	LASSI[c]	First semester GPA[a]	Correlational analysis	Only the Will component was statistically significant in predicting GPA.
Kus-Patena[20]	105	LASSI[a]	semester GPA[a], overall GPA[a]	ANOVA	Gain score differences were found to be statistically significant for the dependent variables of Time Management and Motivation.

[a] Continuous variable, [b] Categorical variable, [c] Composite variable

Key: LASSI = Learning and Study Strategies Inventory; PCAT = Pharmacy College Admission Test; MCAT = Medical College Admission Test; UGPA = Undergraduate GPA; NBME = National Board of Medical Examiners; NBCE = National Board of Chiropractic Examiners
